# Macrocyclic Drugs and Synthetic Methodologies toward Macrocycles

**DOI:** 10.3390/molecules18066230

**Published:** 2013-05-24

**Authors:** Xufen Yu, Dianqing Sun

**Affiliations:** Department of Pharmaceutical Sciences, The Daniel K. Inouye College of Pharmacy, University of Hawai’i at Hilo, 34 Rainbow Drive, Hilo, HI 96720, USA; E-Mail: xufen@hawaii.edu

**Keywords:** macrocycles, macrocyclization, macrocyclic drugs, natural products, retrosynthesis, methodology, macrolactonization, macrolactamization, transition-metal catalyzed cross coupling, ring-closing metathesis, click chemistry

## Abstract

Macrocyclic scaffolds are commonly found in bioactive natural products and pharmaceutical molecules. So far, a large number of macrocyclic natural products have been isolated and synthesized. The construction of macrocycles is generally considered as a crucial and challenging step in the synthesis of macrocyclic natural products. Over the last several decades, numerous efforts have been undertaken toward the synthesis of complex naturally occurring macrocycles and great progresses have been made to advance the field of total synthesis. The commonly used synthetic methodologies toward macrocyclization include macrolactonization, macrolactamization, transition metal-catalyzed cross coupling, ring-closing metathesis, and click reaction, among others. Selected recent examples of macrocyclic synthesis of natural products and druglike macrocycles with significant biological relevance are highlighted in each class. The primary goal of this review is to summarize currently used macrocyclic drugs, highlight the therapeutic potential of this underexplored drug class and outline the general synthetic methodologies for the synthesis of macrocycles.

## 1. Introduction

Macrocyclic motifs are commonly found in natural products and pharmaceutical molecules; and thus provide privileged scaffolds for medicinal chemistry programs in modern drug discovery [1,2]. From a historic and clinical point of view, macrocyclic molecules have had an enormous impact on the fields of chemistry, biology, and medicine [1,3,4,5]. Many naturally occurring macrocycles have been successfully introduced to the clinic; as such, macrocyclic natural products continue to serve as invaluable starting points and to drive and inspire organic and medicinal chemists to discover new and better drugs [1,6,7]. Different from synthetic small molecule drugs, characteristics of macrocyclic natural products typically include a 12 or more membered ring architecture and often do not possess the druglike “rule of five” properties [8]. This unique structural feature and conformational flexibility of the macrocyclic ring can offer subsequent functional advantages, e.g., it has the potential of being highly potent as well as being selective when key functional groups interact with biological targets [1]. In addition, from a chemistry point of view, macrocyclic compounds can offer diverse functionality and stereochemical complexity in a conformationally restricted manner. Moreover, macrocycles can demonstrate favorable druglike properties, including good solubility, increased lipophilicity, enhanced membrane penetration, improved metabolic stability, and good oral bioavailability with desirable pharmacokinetic and pharmacodynamic properties [1,2,4].

## 2. Macrocyclic Drugs

**Figure 1:**
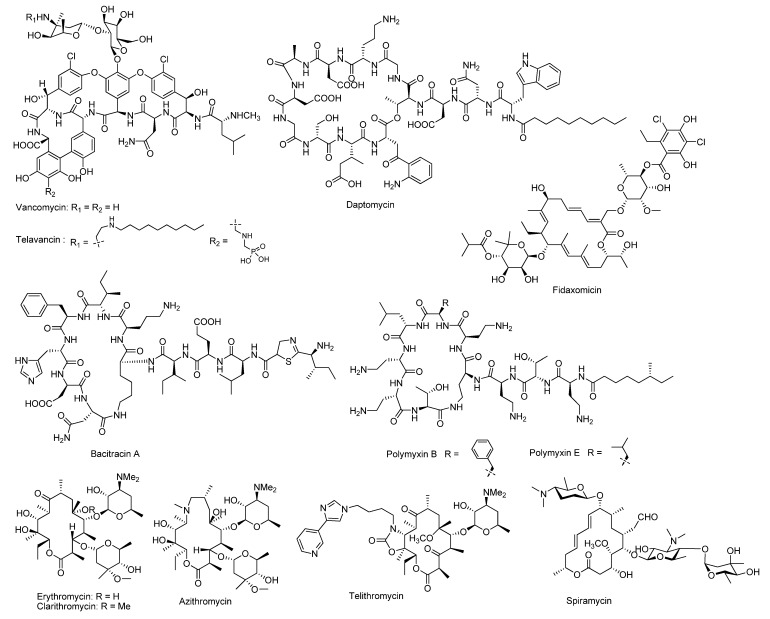
Clinically used macrocyclic antibiotics.

**Figure 2:**
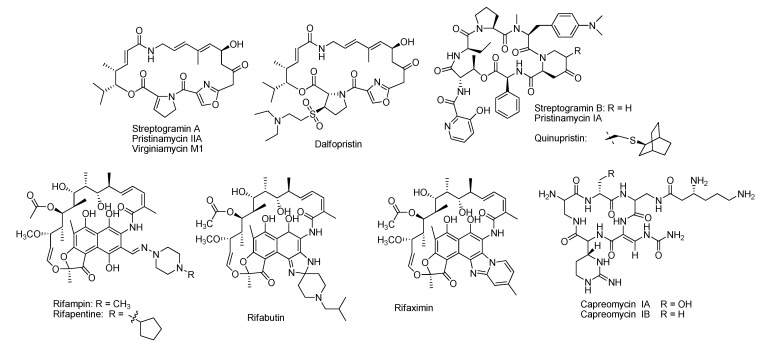
Additional macrocyclic antibiotics.

Although the structural complexity and synthetic intractability limit their pharmaceutical application, macrocycles have broad applications in drug discovery and development; and numerous natural macrocyclic compounds present exceptional therapeutic potential and unrivalled biological activities [1]. Historically, macrocyclic molecules represent a successfully documented drug class in the clinic. In this section we review clinically used macrocyclic drugs and mainly focus on their structural aspect, mechanism of action and primary clinical indication. Notably, the macrocyclic antibiotics (Figure 1 , Figure 2) constitute one of the most successful classes of macrocyclic drugs in clinical practice. Among them, vancomycin is a macrocyclic glycopeptide antibiotics for the treatment of Gram-positive bacterial infections, such as methicillin-resistant *Staphylococcus aureus* (MRSA) and penicillin-resistant *Streptococcus pneumonia* [9,10]. Chemically, vancomycin is a hydrophilic glycopeptide containing a glycosylated hexapeptide chain and aromatic rings cross linked by aryl ether bonds into a rigid molecular framework. It is not orally bioavailable due to poor absorption in the gastrointestinal tract, however, it can be used as an oral antibiotic for the treatment of *C. difficile*-associated diarrhea and enterocolitis caused by *Staphylococcus aureus* [9,10]. In 2009, its synthetic lipoglycopeptide derivative telavancin was approved by the U.S. FDA for the treatment of complicated skin and skin structure infections (cSSSIs) caused by MSSA, MRSA, and vancomycin-susceptible *Enterococcus faecalis*, and *Streptococcus pyogenes*, *Streptococcus agalactiae*, or *Streptococcus anginosus* group [10,11,12]. Mechanistically, this glycopeptide class inhibits the peptidoglycan biosynthesis of bacterial cell wall by binding tightly to D-alanyl-D-alanine portion of cell wall precursor, as well as disrupts cell membrane integrity [10,13,14].

In addition, daptomycin is a new cyclic lipopeptide antibiotic produced from *Streptomyces roseosporus* [15]. It was approved by the U.S. FDA in 2003 for the treatment of cSSSIs caused by susceptible aerobic Gram-positive organisms and *S. aureus* bacteremia caused by MSSA or MRSA [10,16]. Daptomycin rapidly depolarizes bacterial membrane by binding to components of the cell membrane of susceptible organisms and inhibits macromolecular biosynthesis of DNA, RNA, and protein [10,17]. Fidaxomicin, obtained from the fermentation broth of *Dactylosporangium aurantiacum* subspecies hamdenesis, represents the first in a new macrocyclic class of narrow spectrum antibiotics [18,19,20]. It was approved by the U.S. FDA for the treatment of *C. difficile*-associated diarrhea in 2011 [10]. Bacitracin A, generated from the licheniformis group of *Bacillus subtilis*, is a branched cyclic polypeptide broad spectrum antibiotic targeting both Gram-positive and -negative organisms [21,22]. It works by inhibiting the late stage peptidoglycan biosynthesis and disrupting plasma membrane function [23]. Polymyxins A-E belong to an old class of cationic cyclic polypeptide antibiotics that consist of a cyclic positively charged decapeptide with an either 6-methyl-octanic acid or 6-methyleptanoic acid fatty acid side chain. Only polymyxins B and E in this class are used in the clinic, which are primarily used for the treatment of Gram-negative bacterial infections such as *Acinetobacter* species, *Pseudomonas aeruginosa*, *Klebsiella* species, and *Enterobacter* species [10,24,25,26]. Polymyxin B disrupts bacterial membrane integrity by binding to phospholipids in cytoplasmic membranes [10,25].

The prototype macrolide antibiotic erythromycin, bearing a 14-membered macrocyclic lactone motif, was isolated from the fermentation broth of the fungus *Saccharopolyspora erythraea* and used for the treatment of susceptible bacterial infections [27,28]. Clarithromycin, a semisynthetic derivative of erythromycin with a 6-methoxyl ether functionality and improved acidic stability, is an effective macrolide antibiotic for the treatment of chronic bronchitis and erysipelas [29,30]. Azithromycin, a 15-membered expanded ring derivative of erythromycin, is another advanced and effective antibacterial agent in this macrolide class [29,30]. Telithromycin, the first ketolide antibiotic bearing a 14-membered lactone ring and an interesting alkyl-aryl side chain linked with a cyclic carbamate moiety, was approved by the U.S. FDA in 2004 and is used for the treatment of mild-to-moderate community-acquired pneumonia [10,30,31]. This class of macrolide antibiotics exerts its antibacterial action by binding to the 50S subunit of the bacterial ribosome resulting in the inhibition of RNA-dependent protein synthesis [10,32]. Spiramycin is another glycomacrolide antibiotic which is currently not available in the U.S.. It inhibits bacterial growth of susceptible organisms with unknown mechanism of action; and is used for the treatment of bacterial infections of the respiratory tract, buccal cavity, skin and soft tissues due to susceptible organisms [10,33].

As shown in Figure 2, the streptogramin family represents another important class of naturally occurring macrocyclic antibiotics, which includes streptogramin A, streptogramin B, quinupristin, and dalfopristin [34]. This chemical class functions as bacterial protein synthesis inhibitors [34]. Structurally, the streptogramin group A has a 23-membered unsaturated macrolactone with peptide bonds, while the streptogramin group B belongs to a cyclic hexadepsipeptide class. The combination of quinupristin and dalfopristin is used synergistically for the treatment of cSSSIs caused by MSSA or *Streptococcus pyogenes* [10,35].

Rifamycin and its derivatives constitute another notable class of antibacterial agents. The rifamycin antibiotic family includes rifampin, rifapentine, rifabutin, and rifaximin. Chemically, this class consists of a 25-membered macrolactam ring bearing a naphthalenic aromatic moiety connected to an aliphatic chain. Mechanistically, this antibacterial class inhibits bacterial RNA synthesis by binding to the β-subunit of DNA-dependent RNA polymerase [36] and it is primarily used for the treatment of tuberculosis except that rifaximin is clinically used for the treatment of traveler’s diarrhea caused by noninvasive strains of *E. coli* [10]. In addition, capreomycin (administered as a mixture of capreomycin 1A and 1B) is a strongly basic and cyclic polypeptide antibiotic, which is used in the second line TB regimens for the treatment of multi-drug resistant tuberculosis (MDR-TB) in conjunction with other antibiotics [10,37].

**Figure 3:**
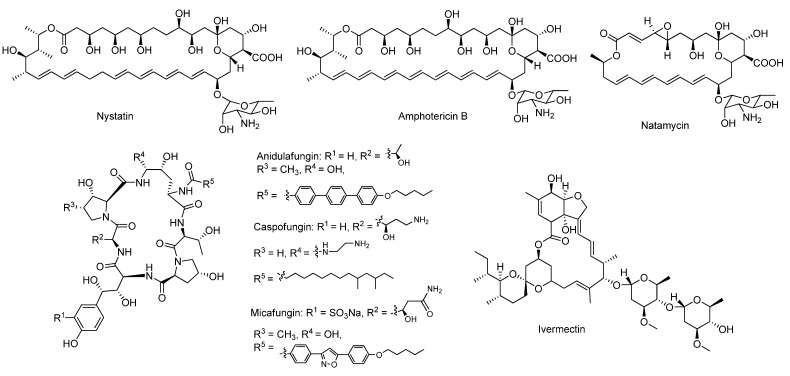
Clinically used macrocyclic antifungal and antiparasitic agents.

Macrocyclic antifungal agents are illustrated in Figure 3. Nystatin, amphotericin B, and natamycin belong to a chemical class of polyene antifungal drugs, which structurally consists of a macrocyclic lactone scaffold; a hydrophilic region containing multiple OH groups, a COOH functionality, and an aminosugar moiety; and a hydrophobic region containing a chromophore of the 4–7 conjugated double bond system. This naturally occurring antifungal class works by binding to ergosterol in fungal cell membrane and thus disrupting fungal membrane function [38,39]. Nystatin, the first clinically used agent in this polyene class, displays potent activity for invasive *Candida* infection; however, it can only be used topically due to its severe toxicity for systemic use [10]. In contrast, amphotericin B is used parenterally for the treatment of severe systemic and CNS fungal infections caused by susceptible fungi [10]. Natamycin is the only topical ophthalmic antifungal agent approved by the U.S. FDA for the treatment of blepharitis, conjunctivitis, and keratitis caused by susceptible fungi (*Aspergillus*, *Candida*, *Cephalosporium*, *Fusarium*, and *Penicillium*) [10].

Structurally, antifungal echinocandins belong to a lipopeptide chemical class, which includes a large cyclic hexapeptide linked to a long fatty acid tail or lipophilic side chain. The echinocandin family includes anidulafungin, caspofungin, and micafungin and is used parenterally for the treatment of candidemia, other forms of *Candida* infections, and invasive *Aspergillus* infections [10,40,41,42]. This drug class demonstrates antifungal activity by inhibiting 1,3-β-D-glucan synthase, an important target in the fungal cell wall biosynthesis [39,40].

On the other hand, macrocycles have also been used as antiparasitic agents. One such example, ivermectin, bearing a 16-membered macrocyclic ring, is an effective antiparasitic and anthelmintic agent for the treatment of strongyloidiasis of the intestinal tract and onchocerciasis, as well as the topical treatment of head lice (Figure 3) [10,43,44]. Ivermectin binds to glutamate-gated chloride ion channels with high selectivity and strong affinity in invertebrate nerve and muscle cells, which ultimately leads to the death of the parasite due to increased permeability of cell membranes to chloride ions and subsequent hyperpolarization of the nerve or muscle cell [10,43].

**Figure 4:**
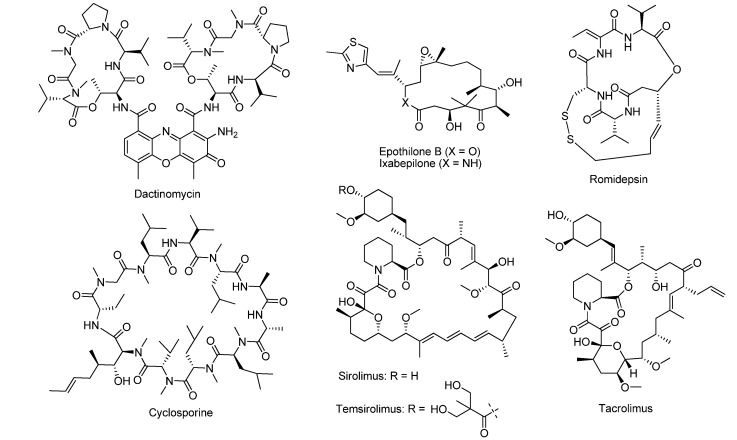
Macrocycles used as cancer chemotherapeutic and immunosuppressant agents.

Macrocyclic anticancer chemotherapeutic agents are shown in Figure 4. As one of the older chemotherapy drugs, dactinomycin, isolated from soil bacteria of the genus *Streptomyces*, is a cyclic polypeptide intravenous antibiotic with anticancer activity [45]. It binds to DNA and causes subsequent inhibition of RNA synthesis and is used in the treatment of Wilm’s tumor, gestational trophoblastic neoplasia and rhabdomyosarcoma [10]. Epothilone B, a 16-membered polyketide macrolactone with a methylthiazole side chain, exerts its cytotoxic effects through promoting microtubule assembly, interfering with the late G_2_ mitotic phase, and inhibiting cell replication [10]. It has similar mechanistic profile as taxanes but improved solubility and milder side effect and become a new class of anticancer drugs for the treatment of metastatic or locally-advanced breast cancer (refractory or resistant) [10,46]. The semisynthetic macrolactam analogue ixabepilone of epothilone B is used for the treatment of advanced breast cancer [47]. In addition, romidepsin, a histone deacetylase (HDAC) inhibitor generated from the bacteria *Chromobacterium violaceum*, is an antineoplastic prodrug for the treatment of refractory cutaneous T-cell lymphoma and refractory peripheral T-cell lymphoma [10,48].

Macrocycles have also been clinically used as immunosuppressant agents, one such example, the cyclic polypeptide cyclosporine inhibits the production and release of interleukin-2 (IL-2), inhibits IL-2-induced activation of resting T-lymphocytes and thus inhibits T cell-mediated immune responses [10,49]. It is frequently used to prevent rejection in organ transplant recipients [10]. Another macrolide lactone class of immunosuppressive agents includes sirolimus (rapamycin) [50] and tacrolimus. Similar to cyclosporine, this macrolide class can also be used in organ transplantations to prevent organ rejection by inhibiting the response to IL-2 or the secretion of IL-2, and subsequently blocking activation of T and B cells [10,51]. However, mechanistically, sirolimus inhibits T-lymphocyte activation and proliferation in response to antigenic and cytokine stimulation and inhibits antibody production [10]. In contrast, temsirolimus, a derivative of sirolimus, was approved by the U.S. FDA in 2007 and is used in the treatment of advanced renal cell cancer [10,52]. Temsirolimus and its active metabolite, sirolimus, function as targeted inhibitors of mTOR (mammalian target of rapamycin) kinase activity [10,52].

**Figure 5:**
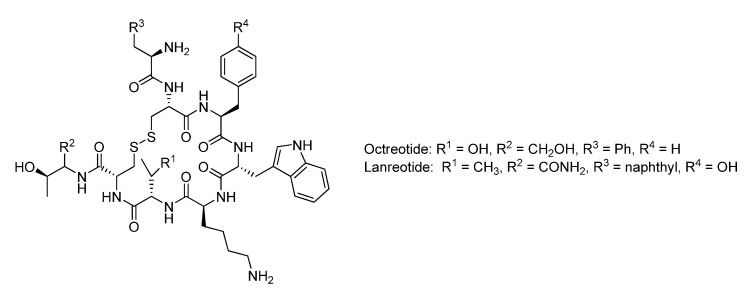
Macrocyclic agents related to pituitary disorders.

Somatostatin, a 38-membered macrocyclic peptide hormone, regulates the release of human growth hormone from the pituitary. The synthetic cyclic octapeptide surrogates octreotide and lanreotide were subsequently developed to mimic the pharmacological activity of endogenous somatostatin (Figure 5) [53,54]. Notably, octreotide exhibits more potent inhibition of growth hormone, glucagon, and insulin relative to somatostatin. Similarly, lanreotide also displays a greater affinity for somatostatin receptors and has a much longer half life than somatostatin [10,54]. Both octreotide and lanreotide are used for the treatment of acromegaly; octreotide is also indicated for the treatment of severe diarrhea and flushing associated with carcinoid syndrome [10,55].

## 3. Chemical Methodologies for the Construction of Macrocycles

Fascinated by intriguing biological activity and inspired by intractable synthetic complexity of naturally occurring macrocycles, much effort has been devoted to explore highly efficient and superior synthetic methods for the preparation of macrocycles [56,57,58,59]. Among macrocyclization methodologies, macrolactonization, macrolactamization, transition metal catalyzed coupling reaction, ring-closing metathesis, and click chemistry represent the most efficient and commonly used synthetic approaches for macrocyclization.

### 3.1. Macrolactonization and Macrolactamization

By far, macrolactones and macrolactams constitute a major part of naturally occurring macrocycles, which mediate diverse biological activities [60]. For the synthesis of macrolactones, many reports regarding efficient macrocyclization have been published. Very recently, Campagne and coworkers provided an excellent and systematic review regarding macrolactonization strategies toward the total synthesis of natural products [61]. In general, the most frequently used and attractive cyclic approaches still come to the direct lactonization of acids and alcohols (seco-acids) using various activation schemes [61]. Briefly, the activation strategies can be classified: (1) activate the acid group to increase electrophilicity of the carbonyl group; (2) convert the alcohol functionality into a preferred leaving group which could be easily attacked by a carboxylate anion; (3) activate both the reacting groups simultaneously using a double activation approach [62,63].

For the activation of the carboxylic acid group, one of the most widely used methods is to use a thioester to facilitate macrolactonization. Corey and Nicolaou reported the first macrolactonization via thioesterification of hydroxyl acid [62]. On the basis of Corey and Nicolaou’s thioester procedure, more advanced protocols and reagents were subsequently developed including Corey and Clark [64], Corey and Brunelle [65], Schmidt [66], and Wollenberg [67] to improve the esterification efficiency. Instead of forming a thioester, Mukaiyama [68] introduced 1-methyl-2-chloropyridinium iodide and 2-chloro-6-methyl-1,3-diphenylpyridinium tetrafluoroborate; and Venkataraman [69] used cyanuric chloride to the macrolactonization reactions. Another approach to activate the acid group is to use 2,4,6-trichlorobenzoyl chloride (Yamaguchi reagent), Yamaguchi macrolactonization remains an attractive approach in the syntheses of macrocycles [70]. Considering the high basicity of DMAP and high reaction temperature of Yamaguchi macrolactonization, a modified Yamaguchi procedure was developed by Yonemitsu [71]. Taken together, these diverse activating reagents and methodologies could complement each other to achieve optimal and effective reaction conditions for macrolactonization.

**Scheme 1:**
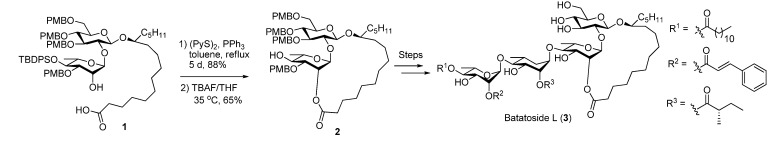
Total synthesis of batatoside L.

Corey and Nicolaou and other alternative methods have been used in a substantial number of synthetic applications including macrolide antibiotics [72]. For example, recently, Yang and coworkers [73] synthesized batatoside L [74,75], bearing a 18-membered macrolactone framework, using Corey-Nicolaou macrolactonization approach. As illustrated in Scheme 1, the construction of the macrolactone core structure **2** was achieved by adopting the Corey-Nicolaou macrolactonization approach from glycosidic acid **1**. This macrocyclization took place in a highly diluted toluene solution (0.75 mM). Batatoside L (**3**) could be generated from glycosylation of heterodisaccharide macrolactone **2** and exocyclic dirhamnose trichloroacetimidate, following appropriate deprotections.

**Scheme 2:**
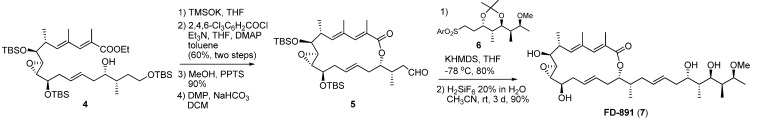
Total synthesis of FD-891.

To date, there are numerous applications in the total synthesis of natural products employing Yamaguchi macrolactonization protocol. One such example, FD-891 (**7**) [76,77,78], a novel 16-membered macrolide antibiotic isolated from the fermentation broth of *Streotomyces graminofaciens A-8890* [79,80], was recently synthesized by Yadav and coworkers by applying Yamaguchi lactonization procedure [81]. As outlined in Scheme 2, hydrolysis of the ethyl ester **4** generated seco-acid, which readily lactonized under standard Yamaguchi conditions to produce macrolactone in 60% yield. Following selective cleavage of the primary TBS protecting group and Dess-Martin oxidation, the aldehyde **5** was obtained. Then, Julia-Kocienski olefination of **5** and sulfone fragment **6** could lead to the final product FD-891 (**7**).

**Scheme 3:**
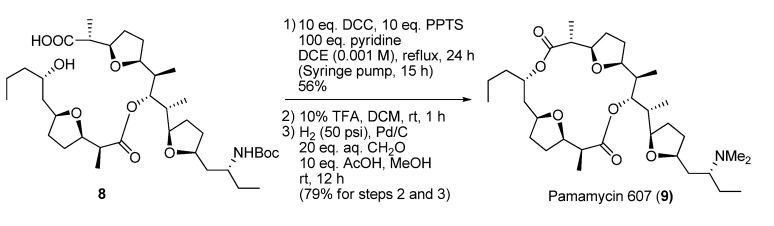
Keck-Boden esterification toward the total synthesis of pamamycin 607.

Another prevalent activating reagent is dicyclohexylcarbodiimide (DCC), which was identified and applied in macrolactonization by Woodward [82]. Then Keck and Boden found DMAP-HCl or other proton acids could enhance macrocyclization efficiency by eliminating undesired *N*-acylurea.[83] Subsequently, the Keck-Boden procedure has a broad application in the total syntheses of macrolactones. For example, in the total synthesis of pamamycin 607 (**9**), the important step was taken by Keck-Boden macrolactonization of seco-acid precursor **8**, which could not be cyclized by Corey-Nicolaou, Mukaiyama, and Yamaguchi-Yonemistu procedures (Scheme 3) [84].

**Scheme 4:**
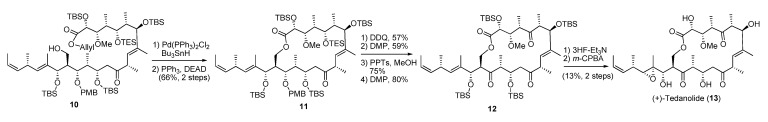
Mitsunobu macrolactonization for the total synthesis of (+)-tedanolide.

Compared to the methods of activating acid groups, the corresponding activation of seco-acid alcohol represents another alternative strategy for macrocyclization. One of the most useful methodologies in total synthesis is the Mitsunobu reaction by treating with diethylazodicarboxylate (DEAD) and triphenylphosphine (PPh_3_) [85]. Mitsunobu lactonization is a popular strategy and used frequently in the total syntheses of macrocyclic natural products [85]. As an example, recently the Kalesse group successfully applied Mitsunobu reaction in the total synthesis of (+)-tedanolide (**13**), which was isolated from the sponge *Tedania ignis* (Scheme 4) [86]. Upon the treatment of palladium catalyzed deprotection of allyl ester **10**, the seco-acid precursor was obtained, followed by Mitsunobu lactonization to facilitate the formation of cyclic **11**. (+)-Tedanolide (**13**) could be obtained following sequential deprotection and oxidation steps. In this case, it is noteworthy that Mitsunobu reaction was much more efficient to complete the lactonization than other methods including Keck-Boden and Yamaguchi esterification.

Other activation methods of alcohols could be accessed via transforming the hydroxyl functionality into a better leaving group, such as halide, mesylate and sulfoniums [61]. In the presence of a base, macrolactonization could be achieved.

**Scheme 5:**
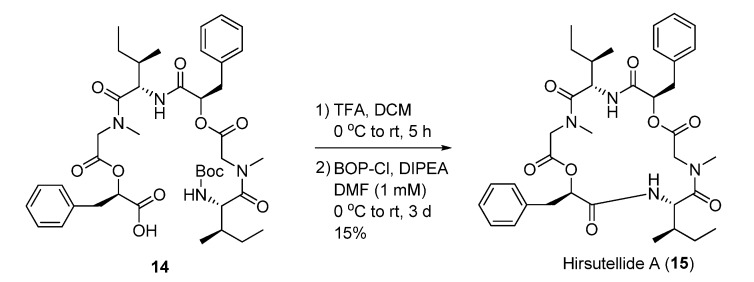
Macrolactamization toward the total synthesis of hirsutellide A.

Compared to macrolactones, the macrolactam scaffold is much less commonly present in many natural products and peptides. Among many methods for synthesizing lactams, the most common and efficient approach is to react an amino group with activated carboxylic acid moiety. Notably, some synthetic methods for activating seco-acid groups to facilitate the formation of active ester in macrolactonization are also applicable to macrolactamization. For instance, the total synthesis of hirsutellide A (**15**) was achieved following a key macrolactamization step of **14** involving the phosphorus activating reagent BOP-Cl and DIPEA in a highly diluted DMF solution (1.0 mM) (Scheme 5) [87].

There are two general ways to activate the carboxylic acid group in macrolactamization. (1) Add uranium-derived reagents or DMT-MM [88] to result in the active ester; (2) use a mixture of a carbodiimide and an additive, such as HOBt, HBTO and HOPO, this strategy could generate the activated ester *in situ*.

**Scheme 6:**
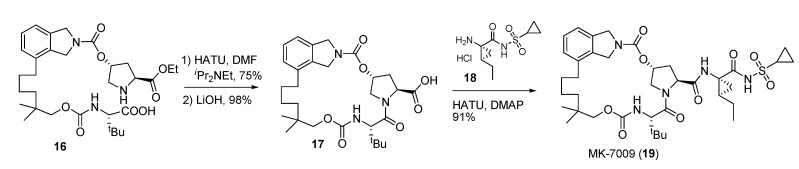
Macrocyclic synthesis of vaniprevir (MK-7009).

Overall, these synthetic methods and reagents are highly efficient and have many applications in the synthesis of macrolactams. For example, vaniprevir (MK-7009), a protease inhibitor of hepatitis C virus (HCV), has been investigated widely and various tactics of synthesis have been developed [89]. Recently, the Song group systematically studied the synthesis of MK-7009 using diverse substrates and conditions [90]. Using HATU as the catalyst, macrolactamization proceeded in 75% yield. Subsequent hydrolysis reaction under LiOH provided **17** in excellent yield. Final condensation of carboxylic acid **17** with amino fragment **18** by employing activating agent HATU under basic conditions afforded vaniprevir (MK-7009) in 91% yield (Scheme 6). 

### 3.2. C-C, C-O, and C-N Coupling Reactions

Over the past decades, transition metal catalyzed cross coupling reactions have become a striking tool for creating new C-C, C-O or C-N bonds. From a synthetic perspective, the diversity of coupling reactions provides a considerable broad stage for enormous applications in organic synthesis [91]. The convenience of constructing new C-C, C-O, C-N and other carbon-hetero (C-X) bonds makes coupling reactions as powerful and attractive tools in natural product synthesis. Consequently, there are a large number of applications in the syntheses of naturally occurring macrocycles.

#### 3.2.1. C-C Bond Formation

**Scheme 7:**
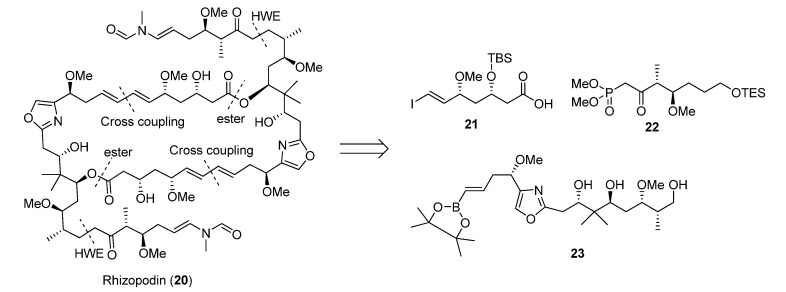
Retrosynthesis of rhizopodin.

Rhizopodin (**20**), isolated from the culture broth of the myxobacterium *Myxococcus stipitatus*, exhibited antifungal and antiproliferative cytotoxicity against a panel of cancer cell lines [92,93]. It possesses a C_2_-symmetric 38-membered macrolide ring containing 18 stereogenic centers. Due to its unique and intriguing structure, this natural product target has aroused considerable interest in synthetic community [94,95,96,97,98,99,100,101]. As outlined in retrosynthetic analysis, the target molecule could be disconnected into three similar fragments **21**–**23** by sequential cross coupling reaction, ester formation reaction and Horner-Wadsworth-Emmons (HWE) coupling/hydrogenation reaction (Scheme 7) [95].

**Scheme 8:**
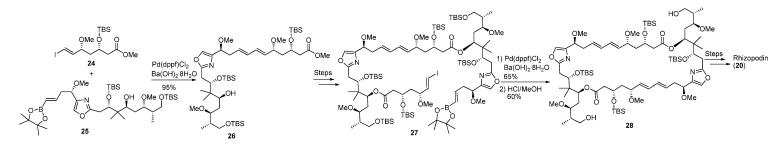
Suzuki cross coupling for macrocyclization of rhizopodin.

The convergent synthetic strategy was initiated by coupling vinyl iodide **24** with the boronate synthon **25** via Suzuki cross coupling in the presence of palladium catalyst. The fragment **27** can be obtained from **26** following two Yamaguchi transformations. Then, Suzuki macrocyclization underwent smoothly to allow the generation of the C_2_-symmetric macrocyclic core **28**, followed by HWE coupling and hydrogenation to install the remaining side chains to complete the total synthesis of rhizopodin (**20**) (Scheme 8) [95].

**Scheme 9:**
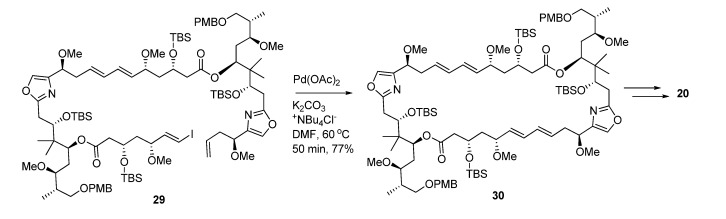
Heck reaction as a key macrocyclization step for the total synthesis of rhizopodin.

Similarly, the Menche group applied Heck reaction as the final macrocyclization key step to realize the total synthesis of rhizopodin (**20**) in a highly concise and efficient way (Scheme 9) [94]. 

It is noteworthy that other coupling reactions between a carbonyl group and a nucleophile could lead to the formation of the C-C bond during the total synthesis, such as Julia-Kocienski coupling, Wittig reaction, Grignard reaction and Nozaki-Hiyama-Kishi reaction. Recently, Andrade and coworkers reported the total synthesis of (−)-4,8,10-tridesmethyl telithromycin (**35**), an analogue of antibiotic erythromycin A [102].

**Scheme 10:**
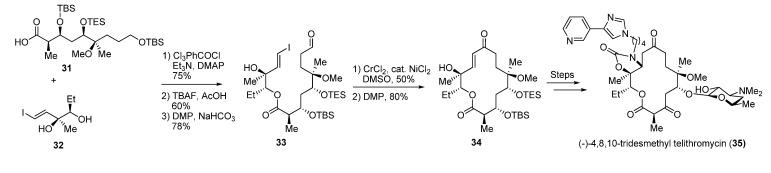
Total synthesis of (−)-4,8,10-tridesmethyl telithromycin.

Yamaguchi esterification of fragments **31** and **32**, followed by deprotection and oxidation reactions, gave the corresponding precursor **33** for macrocyclization. Then, the Nozaki-Hiyama-Kishi reaction was followed and realized the macrocyclization under the treatment of CrCl_2_ and a catalytic amount of NiCl_2_ in DMSO. Following sequential transformations, final (−)-4,8,10-tridesmethyl telithromycin (**35**) could be obtained (Scheme 10).

**Scheme 11:**
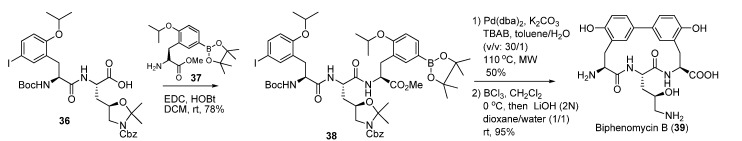
MW-assisted intramolecular Suzuki-Miyaura cross coupling in the synthesis of biphenomycin B.

Tripeptide biphenomycin B (**39**), a macrocyclic natural product, was isolated from the culture broth of *Streptomyces filipinensis* and *S. griseorubuginosus*, and exhibited good antibacterial activities against Gram-positive and β-lactamase resistant bacteria [103,104]. Zhu and coworkers reported the total synthesis of **39** by applying microwave-assisted intramolecular Suzuki-Miyaura cross coupling as a key step for macrocyclization [105]. The coupling reaction was initiated by coupling the amino acid **37** with the free acid form of dipeptide **36** under EDC/HOBt to yield the tripeptide **38**. Final macrocyclization step was completed by MW-assisted intramolecular Suzuki-Miyaura cross coupling in the presence of palladium catalyst. After removing protecting groups, biphenomycin B (**39**) was afforded (Scheme 11).

**Scheme 12:**
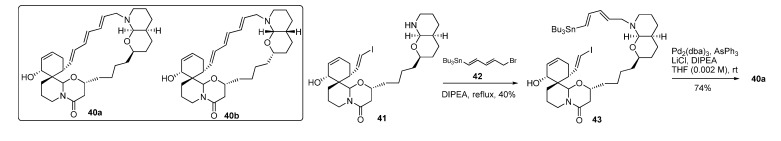
Stille cross coupling as a key step for the total synthesis of ‘Upenamide.

In 2013, Taylor group reported the total synthesis of ‘Upenamide via Stille cross coupling as the key step to construct the cyclic core structure [106]. The proposed structures of these macrocyclic diamine alkaloids **40a** and **40b** consist of unusual tricyclic spirooxaquinolizidinone and octahydropyrano[2,3-b]pyridine ring systems. As shown in Scheme 12, N-alkylation was performed by reacting **41** with stannylpentadienyl bromide **42** under reflux in the presence of Hünig’s base, and the precursor **43** for macrocyclization was obtained in 40% yield. Subsequent intramolecular Stille coupling reaction of **43** was carried out under high dilution conditions (0.002 M) with Pd_2_(dba)_3_, AsPh_3_, LiCl and Hünig’s base in THF to give **40a** in 74% yield.

**Scheme 13:**
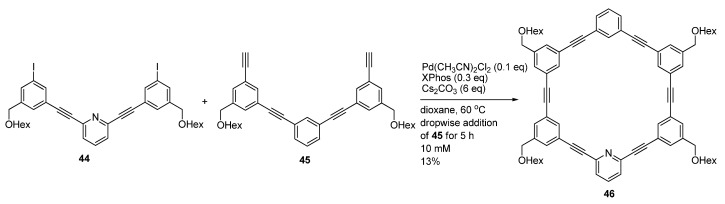
Double Sonogashira cross coupling reactions for the synthesis of SPM.

In host-guest chemistry, shape-persistent macrocycles (SPM) are important scaffolds, which are often derived from arylene and ethynylene units [107]. One of efficient methods for SPM synthesis is via double Sonogashira cross coupling reaction between aryl halides (I, Br) and terminal alkynes. Saito and coworkers prepared a SPM with a single pyridine unit by applying this method. Under the treatment of catalyst Pd(CH_3_CN)_2_Cl_2_, ligand XPhos, and Cs_2_CO_3_ in dioxane, the terminal alkyne **45** was added dropwise to the diluted aryl iodide solution (10 mM) of **44** for 5 h to get the final SPM **46** in 13% yield (Scheme 13) [108].

#### 3.2.2. C-O Bond Formation

In terms of the construction of the C-O bond, the Ullmann reaction has been one of the main strategies in the field of transition metal catalyzed cross coupling reactions [109]. Nowadays, it remains an attractive strategy in the total synthesis of natural products and macrocycles. 

**Scheme 14:**
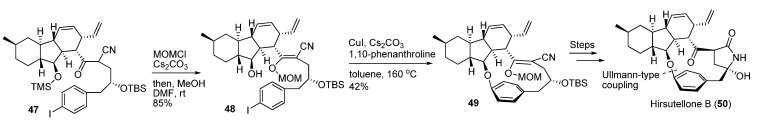
Intramolecular Ullmann-type reaction for the total synthesis of hirsutellone B.

Hirsutellone B (**50**) was isolated from insect pathogenic fungus *Hirsutella nivea* BCC 2594 and showed potent antituberculosis activity [110]. Structurally, it has a highly strained 13-membered cyclic ring and a tricyclic decahydrofluorene skeleton [110]. There are several strategies toward the total synthesis of this challenging macrocycle [111,112,113,114,115,116]. Recently, Uchiro and coworkers accomplished the total synthesis of hirsutellone B by using copper-catalyzed Ullmann-type reaction as a critical cyclic step (Scheme 14) [117]. The MOM-protected enol ether **48** was obtained after reacting **47** with MOMCl in the presence of Cs_2_CO_3_. Then, the 13-membered macrocycle core **49** could be built by intramolecular Ullmann-type etherification of **48** using CuI as catalyst and 1,10-phenanthroline as ligand. Following several transformations, the desired hirsutellone B (**50**) was formed in good yield.

**Scheme 15:**
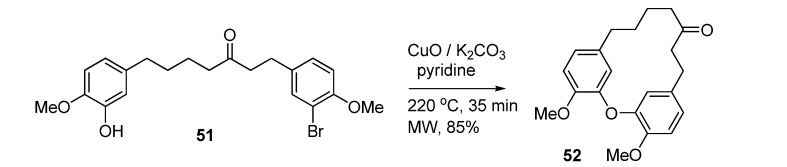
Microwave-assisted intramolecular Ullmann reaction.

For the cyclic ring system, microwave-assisted cyclization by different strategies provides an efficient route to synthesize diverse medium-sized heterocycles [118] and macrocycles [119,120,121,122,123,124]. Recently, Sun’s group reported microwave-assisted intramolecular Ullmann reaction to yield macrocyclic diaryl ether analogues (Scheme 15) [125]. Notably, the MW-assisted Ullmann macrocyclization is much more efficient than previously reported cyclic reaction using sealed pressure tube and conventional heating [126]. To further extend medicinal chemistry effort of this work, Sun *et al.* subsequently synthesized a panel of macrocyclic diarylheptanoid derivatives using a series of aldol condensations, selective hydrogenations, and microwave-assisted intramolecular Ullmann chemistry etc.; and this chemical library was evaluated against a panel of bacterial pathogens. From this study, several reductive amination derivatives with phenethyl- and *n*-hexylamino substituents of **52** demonstrated the most potent antibacterial activity against *M. tuberculosis*, *E. faecalis*, and *S. aureus* with MICs ranging from 12.5–25 μg/mL [127].

#### 3.2.3. C-N Bond Formation

**Scheme 16:**
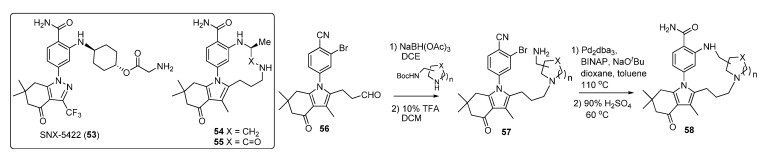
Macrocyclization via Buchwald-Hartwig coupling reaction.

On the other hand, for the construction of the C-N bond, Buchwald-Hartwig coupling reaction represents a prevalent methodology in organic synthesis [128,129]. For example, SNX-5422 (**53**), a glycine pro-drug clinical candidate, was found to display excellent inhibitory activity against Hsp90 in proliferation assay [130]. Inspired by SNX-5422, Zapf and coworkers designed and synthesized a series of macrocyclic *o*-aminobenzamide Hsp90 inhibitors, such as **54** and **55** [131,132]. These derived compounds exhibited potent inhibitory activity of Hsp90 with good solubility and microsomal stability. Recently, they endeavored to synthesize the structural variants of these lead compounds involving amino-based heterocycles into the macrocyclic structure (Scheme 16) [133].

By reacting aldehyde **56** with optically Boc-protected amines, the amine **57** was obtained following N-Boc deprotection under acidic conditions. The final cyclization was performed using Buchwald-Hartwig coupling reaction to efficiently yield an array of macrocycles. The target macrocycles **58** were obtained by transforming the nitrile group into the amide functionality under strong acidic conditions.

As highlighted from selected examples above, the well developed and diverse cross coupling reactions allow macrocyclization performed in a convenient and efficient way in organic synthesis. Evidently, these coupling reactions could also be applied to the total synthesis of naturally occurring products bearing other carbon-hetero bonds.

### 3.3. Ring-Closing Metathesis (RCM) Reaction

The metathesis reaction has emerged as an extremely efficient and powerful tool for the formation of C-C bonds in organic chemistry. Accordingly, its synthetic applications have attracted a great deal of attention from both academia and industry. So far, many reviews concerning metathesis reactions have been published [134,135,136,137,138,139,140,141]. The rapid growth of this field has led to profound synthetic applications of metathesis in advanced organic synthesis. Olefin and alkyne metathesis are transition metal catalyzed reactions which proceed carbon skeleton redistribution and mutual exchange of alkylene or alkynyl groups.

Since the advent of the RCM reaction, a variety of large-ring carbo- and heterocycles and acyclic unsaturated molecules have been efficiently synthesized via this significant class of reactions. Consequently, RCM reactions have played a prominent role in macrocyclic organic synthesis [139,142,143] The following selected examples covering recent total synthesis of macrocycles via diverse RCM strategies are highlighted.

Dactylolide **59**, a novel cytotoxic 20-membered macrolactone, was isolated from a marine sponge of the genus *Dactylospongia* by Riccio and coworkers [144]. On the other hand, its close analogue (−)-zampanolide (**60**), with a large unsaturated framework and an uncommon *N*-acyl hemiaminal side chain, showed potent cytotoxic activities [145,146]. Because of their unique macrolide scaffold and promising biological activity, these macrolide natural products have attracted considerable interest in synthetic organic community and a variety of total syntheses have been realized [147,148,149,150,151,152,153,154,155,156].

**Scheme 17:**
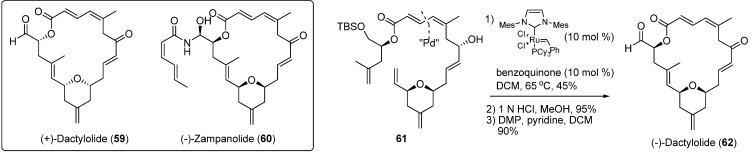
Total synthesis of (−)-dactylolide.

The total synthesis of (−)-dactylolide (**62**) is shown in Scheme 17 [157]. The entire carbon framework of the macrolactone was envisaged to be smoothly assembled via RCM reaction of fragment **61**, resulting in the formation of the trisubstituted double bond. Completion of (−)-dactylolide features the elegant RCM macrocyclic approach. 

**Scheme 18:**
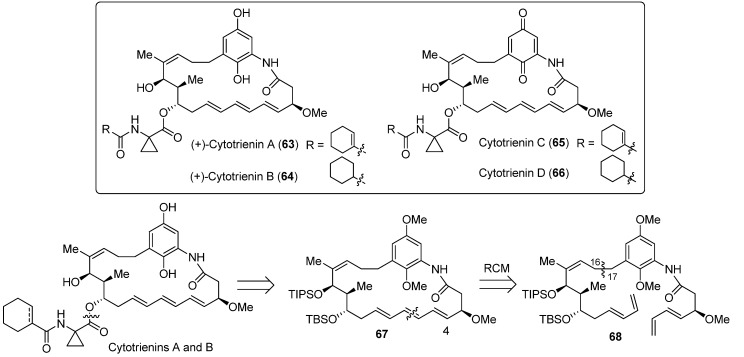
Cytotrienins A-D and representative retrosynthetic analysis of cytotrienins A and B.

Cytotrienins A–D (**63**–**66**, Scheme 18), new ansamycin compounds, possess an unique *E,E,E*-triene motif and four chiral centers, constituting a 21-membered macrocyclic lactam. Based on their challenging structure and promising biological activity, cytotrienins were synthesized using different strategies by the Panek [158], Krische [159], and Hiyashi [160] groups, but the RCM reaction constituted a pivotal step of constructing this macrocyclic lactam from the *E,E,E*-triene motif. Panek commenced the RCM macrocyclization from the bis-1,3-diene **68** fragment (Scheme 18) [161].

**Scheme 19:**
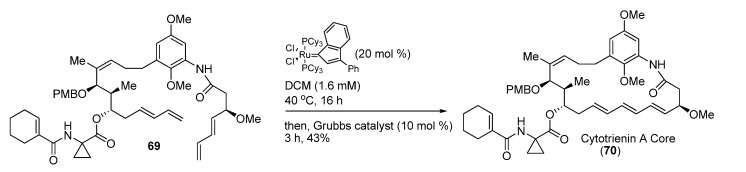
Total synthesis strategy of cytotrienin A core.

On the other hand, Krische and coworkers employed hydrogen-mediated Suzuki-coupling reaction of bromoalkene and pinacol borane to facilitate the formation of the C16-C17 bond (Scheme 19) [159]. Following several transformations, the key precursor bis(diene) **69** was obtained. Treatment of **69** using Grubbs 2nd generation metathesis catalyst in DCM (1.6 mM) afforded the cytotrienin A core **70** in 43% yield.

BILN 2061 (**71a**), a 15-membered macrocycle, is a NS3 protease inhibitor with antiviral effects in humans infected with HCV [162]. It has been synthesized via RCM reaction as the key step for macrocyclization by several groups [163,164,165]. Recently, Wei and coworkers reported a highly convergent and efficient synthesis of BI 201302 (**71b**), a structural variant of BILN 2061 and HCV protease inhibitor, based on the scale-up synthesis of BILN 2061 [166].

**Scheme 20:**
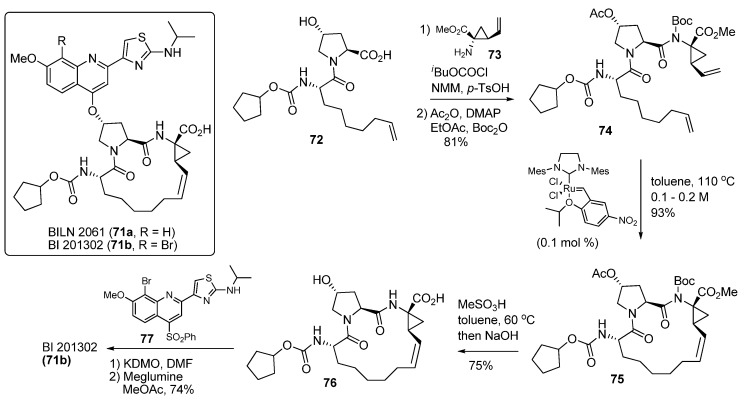
Synthesis of HCV protease inhibitor BI 201302.

As shown in Scheme 20, using the similar starting material for the synthesis of BILN 2061, the synthesis of BI 201302 was initiated by the amide coupling reaction **72** and **73**. Different from the synthesis of BILN 2061, N-Boc protection was introduced to the synthesis to give the N-Boc protected **74**, which could change the initiation site of the RCM reaction by interrupting the coordinative stabilization by the ester group and increase the reaction concentration dramatically to 0.1–0.2 M under the treatment of Grela catalyst (0.1 mol %). The RCM reaction was instantaneously initiated and furnished the desired RCM product **75** in excellent yield. Another improvement is to employ S_N_Ar reaction of **76** and **77** in the final step instead of using S_N_2 to make the reaction less lengthy and more economical [166].

Nakadomarin A, consisting of an unprecedented 8/5/5/5/15/6 ring system, was isolated from the marine sponge *Amphimedon* sp. by Kobayashi and coworkers [167]. The interesting biological activities and complex ring-fused structure have attracted extensive interest in the total synthesis of nakadomarin A, some strategies involved expedient RCM reactions to build the macrocyclic ring [168,169,170,171].

**Scheme 21:**
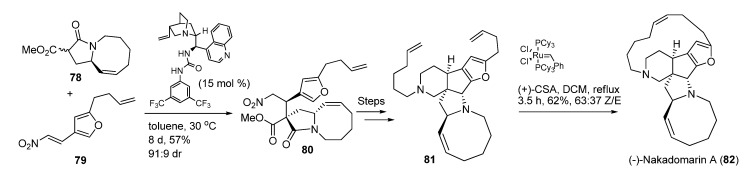
Stereoselective controlled total synthesis of (−)-nakadomarin A.

Nishida and coworkers reported the first total synthesis of nakadomarin A in 2003 [169]. Kerr’s group later devised a shortened synthetic route [171]. Both strategies gave the product with poor *E*/*Z* selectivity (*E* isomer was the major product from NMR). However, Dixon and coworkers started from 8,5-bicyclic pro-nucleophile **78** and furanyl nitro olefin fragment **79** to deliver nitro ester **80** in a stereo-controlled way utilizing bifunctional organocatalysis (Scheme 21) [172]. Following several transformations of nitro ester **80** and a sequence of diastereoselective multicomponent nitro-Mannich/lactamization cascade reaction, the precursor core **81** could be obtained. Upon treatment of **81** with Grubbs 1st generation catalyst and CSA, the synthesis of (−)-nakadomarin A (**82**) was achieved by *Z*-selective olefin metathesis (*Z*/*E* = 63:37). To further develop catalyst-controlled stereoselective RCM, Hoveyda and coworkers exploited other catalyst systems to complete the synthesis of macrocyclic natural products with excellent *Z*-selectivity [173,174].

Although RCM reaction has been developed as a great and efficient way to achieve the syntheses of carbocycles, there are still some limitations when this method is applied to sterically hindered or electronically deactivated substrates. As a complementary reaction to traditional RCM, Hoye’s group has developed relay ring-closing metathesis (RRCM) reaction to circumvent the reactivity or selectivity problems and expand the application scope of RCM reaction by modifying the imperfect RCM substrates rationally [175].

**Scheme 22:**
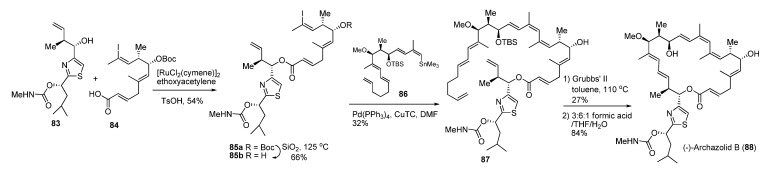
RRCM for the total synthesis of (−)-archazolid B.

By using RRCM reaction, Trauner and coworkers realized the total synthesis of (−)-archazolid B, a 24-membered macrolactone [176]. As outlined in Scheme 22, esterification of **84** with **83** under the catalyst [RuCl_2_(cymene)]_2_ afforded **85a** in 54% yield. For the consideration of reaction selectivity, the precursor **87** for the RCM macrocyclization was designed with a longer chain via Stille cross coupling reaction of **85b** with **86**. Under the treatment of Grubbs’ second generation catalyst, the RRCM reaction of **87** proceeded to afford the macrocycle in 27% yield by releasing cyclopentene; archazolid B was obtained in 84% yield following the *O*-TBS deprotection.

In addition, although there are numerous reports using the RCM reaction to construct complex natural products via the key diene intermediate, heteroatom-substituted olefins have emerged as synthons to access some rare macrocyclic systems. Recently, Evano and coworkers reported the total synthesis of alkaloid paliurine E (**91**) with a 13-membered ring system by employing ene-enamide RCM macrocyclization. Importantly, this work represented the first successful example of applying the ene-enamide RCM toward macrocycle synthesis [177].

**Scheme 23:**
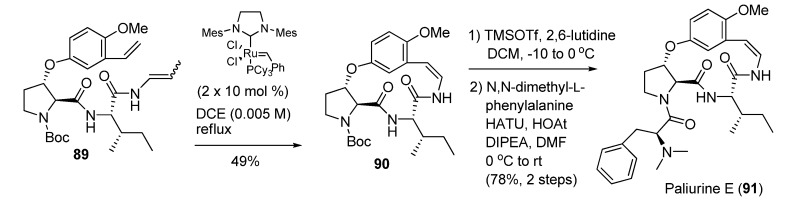
Total synthesis of paliurine E via ene-enamide RCM macrocyclization.

In the course of RCM macrocyclization, paliurine E (**91**) was synthesized from ene-enamide **89** (Scheme 23) [177]. From this work, a dramatic difference was observed based on minor changes on the enamide fragment after treating with Grubbs 2nd generation catalyst. With no substitutents, the enamide fragment could decompose into the amide byproduct (78%) due to its thermal instability and only give the ring-closing product in 8% yield. However, the reaction delivered the desired cyclopeptide core **90** in 49% yield by appending a methyl group on the enamide moiety. The new insights of macrocyclization by ene-enamide RCM reactions feature the flexibility in strategy of total synthesis and increase the usefulness of this method.

**Scheme 24:**
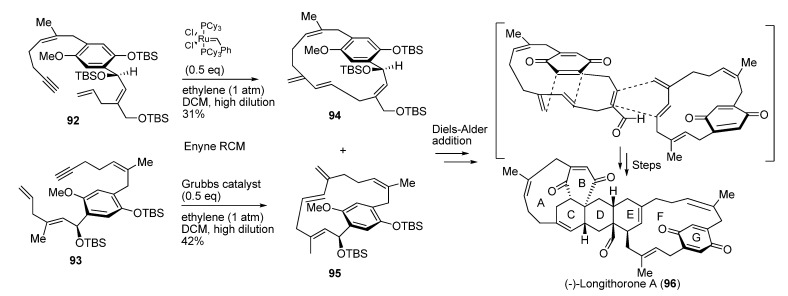
Convergent synthesis of (−)-longithorone A.

Besides alkene metathesis, the enyne-metathesis reaction has also emerged as a particular tool for the access of conjugated 1,3-diene systems from a simple alkene and alkyne through a bond reorganization [178,179]. As such, intramolecular enyne-metathesis reaction represents an extremely useful method for the construction of carbo- and heteromacrocycles. Moreover, the stereoselective issue of intramolecular enyne-metathesis is more requisite than intermolecular enyne-cross metathesis, and requires careful consideration [180,181]. On the other hand, most of the carbene catalysts, which served in alkene-metathesis, can be used in this process to promote enyne-metathesis reactions with high activity and functional group tolerance.

(−)-Longithorone A (**96**) is a cytotoxic marine natural product bearing an unprecedented carbocyclic skeleton [182], and its total synthesis posed a significant challenge to synthetic community. Inspired by the impressive assumption of its biosynthesis, Shair *et al*. developed the synthesis of longithorone A by employing intermolecular Diels-Alder cycloaddition to form ring E and a transannular Diels-Alder reaction to build rings A, C, and D simultaneously [183]. As outlined in Scheme 24, ene-yne metathesis macrocyclization reactions of compounds **92** and **93** using Grubbs 1st generation catalyst afforded the 1,3-disubstituted dienes **94** and **95**, respectively. Subsequent intermolecular Diels-Alder cyclization was performed under mild conditions involving the assistance of stereogenic centers to control atropisomerism. The first application of enyne-metathesis reaction to the construction of macrocycles demonstrated an unique mode of ene-yne metathesis to gear the 1,3-dienes. Collins and coworkers recently reported the macrocyclic en-yne metathesis using highly active Grubbs-Hoveyda catalysts and when exploiting fluoroarene-arene interactions, which could be applied toward the preparation of members of the longithorone family of natural products [184].

As described above, the alkene RCM reactions exemplify an expedient route for macrocyclization, nevertheless, the cycloalkenes formed in a mixture of *E*- and *Z*-geometrical isomers constitute a crucial challenge for this class of reactions. However, alkyne RCM and subsequent stereoselective partial reduction of the nascent triple bond could manipulate the *E*/*Z* stereochemistry of macrocyclic alkenes and has gained a position of increasing significance [185]. Unlike enyne-metathesis, the routine carbene based catalysts could not be used in the corresponding alkyne-metathesis reactions. Recently, Wu and Tamm provided an excellent review regarding the advances in the development of alkyne metathesis catalysts [186].

**Scheme 25:**
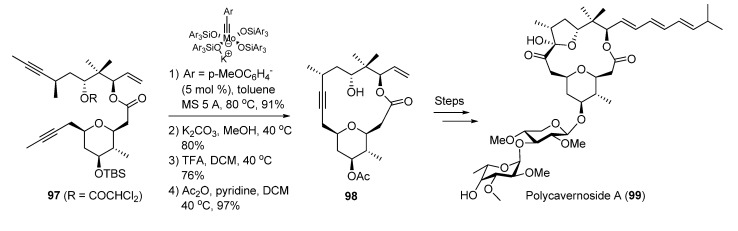
Total synthesis of polycavernoside A.

In this context, alkyne-metathesis ring-closing reaction has been applied widely as an alternative scenario for forging functionalized macrocycles. For example, the naturally occurring marine product polycavernoside A (**99**) with potent bioactivity and intricate structure was synthesized by the Fürstner group via alkyne RCM reaction as a critical macrocyclization step [187]. As outlined in Scheme 25, the orthogonal dichloroacetate diyne **97** was subjected to macrocyclization by intramolecular alkyne RCM reaction under the catalyst molybdenum alkylidyne ate-complex endowed with triarylsilanolate ligands and the macrocycle **98** was obtained after sequential steps. Then following several transformations polycavernoside A could be obtained.

In terms of solid-supported RCM macrocyclization strategy, recently, in the search of insulin-regulated aminopeptidase (IRAP) inhibitors, the Hallberg group identified angiotensin IV (Ang IV, **100**) with potent inhibitory activity with a *K_i_* value of 62.3 nM. Subsequently, they designed and synthesized a series of Ang IV-inspired macrocyclic derivatives with increased steric constraints [188]. The macrocyclic analogues were synthesized by solid-phase supported RCM reaction and the cyclic analogue **101** demonstrated stronger inhibitory activity against IRAP (*K_i_* = 4.1 nM). On the other hand, Hallberg and coworkers designed and synthesized another new series of potent druglike macrocyclic IRAP inhibitors. The compound **102** with a cyclic disulfide bridge exhibited excellent inhibitory activity against IRAP (*K*_i_ = 3.3 nM) [189].

**Scheme 26:**
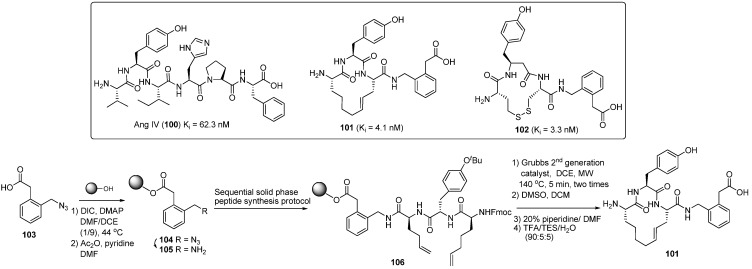
Solid phase supported synthesis of angiotensin IV analogue **101**.

As shown in Scheme 26, solid-phase synthesis of **101** was initiated by loading 2-(azidomethyl)phenylacetic acid **103** to the Wang resin to produce solid supported azide **104**. After reduction and standard solid phase peptide chemistry including coupling with Fmoc-Hag-OH and Fmoc-Tyr(*^t^*Bu)-OH sequentially, the solid supported diene **106** was obtained as the RCM cyclization precursor. Final macrocyclization was performed by using Hoveyda-Grubbs 2nd generation catalyst under MW heating to give the double bond migration product **101** (*E* isomer > 60%) following resin cleavage under acidic conditions. The cyclic **101** could be further transformed into the corresponding reductive variant under Pd/C catalyst and hydrogen.

**Scheme 27:**
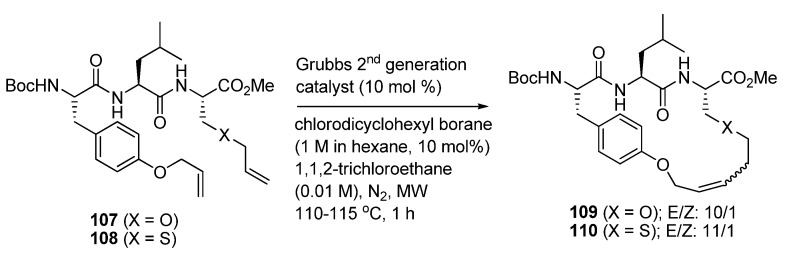
MW-assisted RCM macrocyclization in the syntheses of *β*-strand molecules **109** and **110**.

In addition, MW was also applied to RCM-based macrocyclization in the synthesis of natural products and natural product-like libraries. The Abell group improved the efficiency of final key RCM reaction to afford macrocyclic *β*-strand molecules **109** (*E/Z*: 10:1) and **110** (*E/Z*: 11:1) under MW irradiation combined with Lewis acid (Scheme 27) [190].

Molecular diversity of screening compound collections plays an important role in drug discovery [191,192]. In general, chemical diversity can refer to appendage diversity, functional group diversity, stereochemical diversity, and scaffold diversity [193,194,195]. In the past decade, diversity-oriented synthesis (DOS) emerged as an extremely powerful tool for the generation of diverse natural product-like libraries including macrocycles [191,196,197,198]. As such, DOS-oriented library collections provide invaluable compounds with a significant degree of structural and functional diversity, and could offer an effective strategy to fish out druglike targets [191,199,200]. Furthermore, employing well-established and selective reactions to introduce chemical complexity and diversity, DOS can enable efficient screening of diverse and complex macrocyclic molecular libraries as molecular probes, pharmacological tools, and potential therapeutic agents [201,202].

**Scheme 28:**
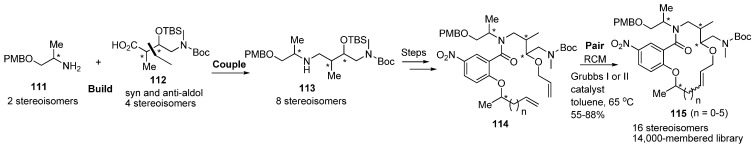
Diversity-oriented synthesis of macrolactam libraries via RCM reaction.

One of the most common schemes used in DOS is the build/couple/pair (B/C/P) approach [203,204,205,206]. For example, by incorporating this strategy, the Marcaurelle group achieved the syntheses of 13- to 18-membered macrolactam libraries with a high degree of diversity via RCM reaction (Scheme 28) [207]. The build step was initiated by diastereoselective adol reaction to obtain **112** with 4 stereoisomers. The subsequent couple step was achieved by coupling amine **111** and amino acid **112** to afford **113** with 8 stereoisomers. After several transformations, compound **114** was produced as the precursor for the last pair step using RCM-based macrocyclizaton. This DOS method provided a feasible and rapid entry to build a complex macrocyclic core library with 14,000 members.

From the view of total syntheses of macrocycles, the alkene and its siblings, such as enyne- and alkyne-metathesis have emerged as extremely powerful tools beyond our imagination over the past decades. RCM macrocyclization features versatility and flexibility of retrosynthetic analysis tactic and produces complex natural products in a spectacular way. It is evident that RCM reactions have demonstrated and revolutionized the methods of total synthesis. Nevertheless, there are still some limitations, such as generally highly diluted metathesis reaction conditions, high catalyst loading, and general low *E*/*Z* selectivity. It is noteworthy that there are various strategies to furnish the *E* and *Z* macrocycles with good selectivity. For example, Young and coworkers have developed a strategy to obtain energetically less favored *Z* macrocycles by attaching a removable silyl group on the olefin motif [208]. Grubbs *et al*. have applied new catalyst systems to achieve the *Z*-selective RCM macrocyclization [209]. In addition, Shrock and Hoveyda groups have used Mo- or W-based monoaryloxide pyrrolide catalyst to realize the *Z*-selective RCM macrocyclization [174]. Combined with emerging schemes to control the outcome of stereochemistry, the utility of the RCM macrocyclization would be greatly enhanced in the course of total synthesis of natural products.

### 3.4. Click reaction

According to the Sharpless defined criteria, click chemistry requires high yield with high stereo- and regioselectivity under mild reaction conditions and excellent functional group tolerance [210]. In general, click reactions can be classified into copper catalyzed azide-alkyne cycloaddition (CuAAC), Diels-Alder cycloaddition, thiol-ene or -yne, and nitroxide radical coupling reactions [210,211,212,213]. The CuAAC click reaction was developed by Sharpless and coworkers in 2001, which involves a modified Huisgen 1,3-dipolar cycloaddition of an alkyne and an azide in the presence of copper (I) catalyst to generate a 1,2,3-triazole product under mild reaction conditions [214,215]. Click chemistry has served as one of the most popular and practical methods in organic synthesis within the last decade [216,217,218,219].

The 1,2,3-triazole heterocyclic motif is commonly found in pharmaceutical and bioactive molecules [220,221,222,223]. Interestingly, this heterocyclic triazole ring possesses similar physicochemical properties with the *trans*-amide functionality so that it could serve as its bioisostere in peptides and peptidomimetics. Many applications have exemplified that compounds with the 1,2,3-triazole linkage can display better biological activity and improved metabolic stability [216,224,225]. Moreover, the click reaction can use a wide range of alkyne and azide substrates and demonstrates excellent tolerance of organic functional groups. In the literature, copper-catalyzed click reaction has also been used extensively in diverse chemical and biological systems as well as in the synthesis of macrocycles [226,227,228]; and thus plays a significant role in organic synthesis and chemical biology [229]. In addition, the click reaction has also been investigated in metal-free conditions [213] and in the synthesis of macrocycles using copper flow reactor technology [230], among others.

**Scheme 29:**
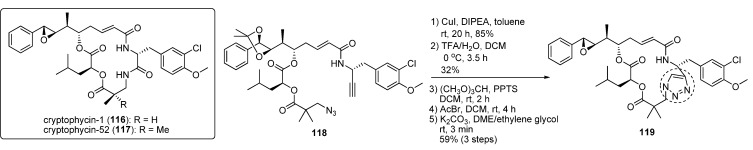
Click reaction for the synthesis of cryptophycin triazole derivative.

As examples, the Sewald group recently replaced the *trans*-amide peptide linkage in the cryptophycin derivatives with 1,4-disubstituted 1*H*-1,2,3-triazole moiety to probe the bioequivalence (Scheme 29) [231]. The cryptophycin triazole analogue **119** could be obtained from **118** via the CuI catalyzed click reaction after sequential steps.

**Scheme 30:**
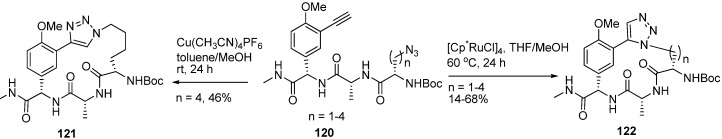
Click reaction for synthesizing vancomycin-inspired mimics.

Similarly, Liskamp and coworkers applied the same strategy to furnish the regioselective synthesis of vancomycin mimics with 1,4- and 1,5-disubstituted triazole-containing macrocyclic tripeptides **121** and **122** by employing Cu(CH_3_CN)_4_PF_6_ and [Cp^*^RuCl]_4_ catalyst, respectively (Scheme 30) [232].

**Scheme 31:**
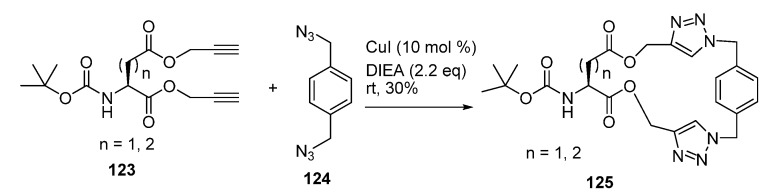
Double copper (I) catalyzed 1,3-dipolar cycloaddition reactions.

Moreover, the Haridas group designed and synthesized novel triazolophanes **125** utilizing double copper (I) catalyzed 1,3-dipolar cycloaddition reactions to construct macrocyclic analogues bearing bis-1,2,3-triazole moieties in one step (Scheme 31) [233].

Among many other applications, Bahulayan and Arun recently reported the synthesis of 12- and 14-membered cyclic peptidotriazoles using a two-step macrocyclization strategy including a four-component reaction and an intramolecular click chemistry [234].

**Scheme 32:**
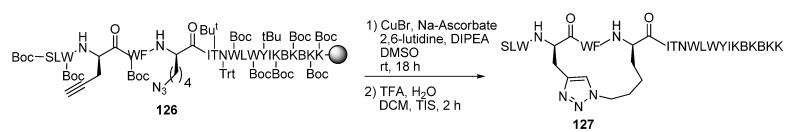
Solid-phase synthesis of representative peptide analogue using CuAAC.

The intramolecular copper catalyzed click cyclization of terminal alkynes and azides also plays an important role in solid-phase supported peptide synthesis [235]. Recently, the Dawson group reported the synthesis of a series of 21 amino acid helical peptides on solid support by employing CuAAC macrocyclization (Scheme 32) [236].

**Scheme 33:**
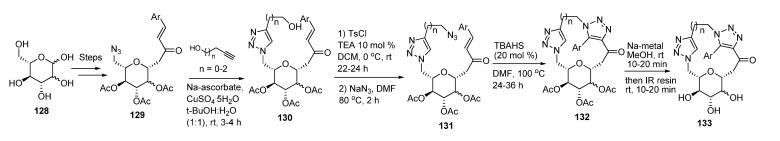
DOS of diverse macrocyclic glycoconjugates.

As illustrated above, the 1,2,3-triazole moiety is an attractive surrogate of the amide group in the peptide backbone due to its remarkable stability and lipophilicity. Development of diverse 1,2,3-triazole macrocyclic peptides holds great potential in advanced drug design and discovery. Very recently, Tripathi and coworkers furnished a series of structurally unique and diverse macrocyclic glycoconjugates by employing a DOS approach [237]. This strategy was outlined based on three steps, including forming a polyfunctional pyran backbone, 1,4-disubstituted triazole and 1,4,5-trisubstituted triazole (Scheme 33). The multifunctional intermediate **129** could be derived from commercially available D-glucose and D-mannose by introducing different aryl groups. The first 1,4-disubstituted triazole **130** was formed by intermolecular click cyclization. Furthermore, after simple transformation, the azide moiety was introduced into the skeleton to yield the second 1,4,5-trisubstituted triazole **132** by click reaction. In this collection, stereochemical and functional diversity is high with five chiral centers and with a variety of different aryl substituents or different lengths of the tether linker between the two triazoles.

## 4. Conclusions

In summary, naturally occurring macrocycles have been successfully used in the clinical practice; new chemotype macrocyclic molecules hold great potential and represent an important class of therapeutic agents. Macrocyclization has thus evolved into an extremely useful and powerful reaction in organic synthesis. There are numerous routes and methodologies leading to the construction of macrocycles. In this review, we first highlight clinically used macrocyclic drugs and then overview the common synthetic methodologies toward macrocycles, which involve macrolactonization/macrolactamization derived from carboxylic acid and intramolecular coupling alcohol and amine partners, transition metal catalyzed cross coupling reactions, RCM reactions, and click chemistry from terminal alkyne and azides. Selected recent applications are highlighted and exemplify these representative synthetic methodologies in macrocyclic organic synthesis.
